# SIRT1, 2, 3 protect mouse oocytes from postovulatory aging

**DOI:** 10.18632/aging.100911

**Published:** 2016-03-10

**Authors:** Teng Zhang, Yang Zhou, Li Li, Hong-Hui Wang, Xue-Shan Ma, Wei-Ping Qian, Wei Shen, Heide Schatten, Qing-Yuan Sun

**Affiliations:** ^1^ Institute of Reproductive Sciences, College of Animal Science and Technology, Qingdao Agricultural University, Qingdao, China; ^2^ State Key Laboratory of Stem Cell and Reproductive Biology, Institute of Zoology, Chinese Academy of Sciences, Beijing, China; ^3^ Department of Reproductive Medicine, Guangdong Women and Children Hospital, Guangzhou, China; ^4^ Department of Reproductive Medicine, Peking University Shenzhen Hospital, Medical Center of Peking University, Shenzhen, Guangdong, China; ^5^ Department of Veterinary Pathobiology, University of Missouri, Columbia, MO 65211, USA

**Keywords:** postovulatory aging, SIRT1, 2, 3, nicotinamide, caffeine

## Abstract

The quality of metaphase II oocytes will undergo a time-dependent deterioration following ovulation as the result of the oocyte aging process. In this study, we determined that the expression of sirtuin family members (SIRT1, 2, 3) was dramatically reduced in mouse oocytes aged in vivo or in vitro. Increased intracellular ROS was observed when SIRT1, 2, 3 activity was inhibited. Increased frequency of spindle defects and disturbed distribution of mitochondria were also observed in MII oocytes aged in vitro after treatment with Nicotinamide (NAM), indicating that inhibition of SIRT1, 2, 3 may accelerate postovulatory oocyte aging. Interestingly, when MII oocytes were exposed to caffeine, the decline of SIRT1, 2, 3 mRNA levels was delayed and the aging-associated defective phenotypes could be improved. The results suggest that the SIRT1, 2, 3 pathway may play a potential protective role against postovulatory oocyte aging by controlling ROS generation.

## INTRODUCTION

Upon luteinizing hormone (LH) surge stimulation, the prophase I oocyte resumes meiosis and undergoes a maturational process involving germinal vesicle breakdown, and extrusion of the first polar body [1]. Following these events, the oocyte once again enters meiotic arrest (now at metaphase II), and remains in this state until fertilization [2, 3]. An optimal window exists in which fertilization of this MII stage oocyte should occur. If no fertilization occurs, with increasing time following ovulation, the MII oocyte undergoes a process of deterioration in vivo and in vitro, referred to as postovulatory aging [4, 5].

Postovulatory aged oocytes display partial cortical granule exocytosis [6, 7] and zona hardening [7]. Additionally, these oocytes commonly exhibit mitochondrial dysfunction [8–11], spindle abnormalities [12], epigenetic changes [13] and loss of chromosomal integrity [12]. As a result, the deterioration associated with postovulatory aging can strongly influence fertilization and subsequent embryo development [14].

Oocyte aging is associated with many deleterious effects, including temperature, cumulus cells, reactive oxygen species (ROS), and others [15]. A gradual accumulation of damage by super-oxide anion and peroxynitrite reactive compounds has been considered as the major mechanism underlying postovulatory aging. Recently, a growing body of evidence has confirmed that the aging process is regulated by a continuous crosstalk between ROS and the sirtuin family. The sirtuins (silent information regulator 2 (Sir2) proteins) are a class of NAD+-dependent deacetylases comprised of seven members in mammals; they are involved in many biochemical processes. The seven members of the mammalian sirtuin family are emerging as key anti-aging molecules and regulators in many diseases. Their ability to regulate systems that control the redox environment has the potential to help counteract oxidative damage. SIRT1 has been shown to be a key player in caloric restriction, which has been shown to increase the lifespan in a variety of organisms [16, 17]. The gene expression of SIRT1 is modulated in response to mild oxidative stress [18]. Oxidative stress has been shown to promote the inactivation of SIRT1 [19]. Previous research has suggested that SIRT1 might be involved in oocyte maturation by regulating the redox state [20]. Meanwhile, SIRT1 has been proved to protect pig oocyte against in vitro aging [21]. The first identified pathway of SIRT1 involved the tumor suppressor p53. As a transcription factor, p53 in response to ROS has been shown to be dependent on SIRT1 deacetylation [22]. A second target of SIRT1 is FOXO3a (forkhead box O 3a), a transcriptional activator of the SOD2 gene which encodes the MnSOD (manganese superoxide dismutase) antioxidant protein [23]. Both SIRT1 and SIRT2 have been proved to deacetylate and activate FOXO3a against oxidative stress [24, 25]. Although the role of SIRT2 has not been characterized as well as that of SIRT1, it does play a regulatory role in modulating oxidative stress. Many studies have confirmed that oxidative stress can result in the up-regulation of both SIRT2 transcript and protein levels [25, 26]. In mitochondria, as the major sirtuin deacetylase, SIRT3 plays a role in the control of ROS generation and amelioration [27]. The generation of mitochondrial ROS has been shown to result in the regulation of both SIRT3 transcript and protein levels [28]. A recent study identified SIRT3 as an important player in modulating ROS homeostasis during mouse oocyte maturation [29]. In addition, SIRT3 also appears to be involved in protecting against stress conditions during fertilization in vitro [30]. NAM acts a non-competitive pan-sirtuin inhibitor by reacting with the ADP-ribose peptideimidate intermediate to reform NAD^+^ protein [31]. A recent study examined the effects of NAM on oocyte meiosis progression [32]. Additionally, NAM causes developmental defects and increases the level of mitochondrial ROS in preimplantation embryos [33].

Although the postovulatory aging phenotype has been well characterized, the underlying mechanisms remain to be discovered. In the present study, we investigated whether SIRT1, 2, 3 play a pivotal role in protecting postovulatory oocytes against oxidative stress and possible alterations linked to postovulatory oocyte aging.

## RESULTS

### Expression of SIRT1, 2, 3 during oocyte aging in vivo and in vitro

**Figure 1:**
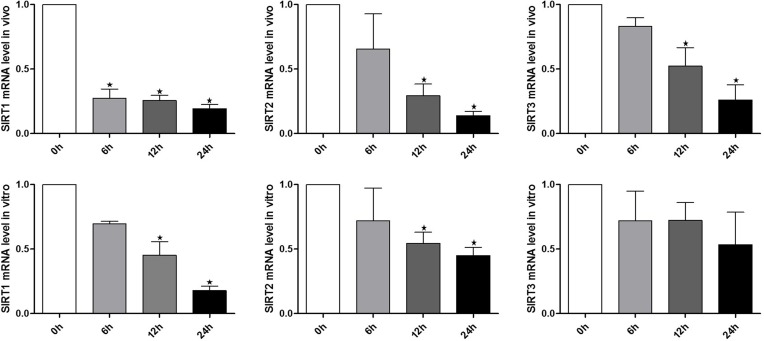
Expression of SIRT1, 2, 3 during oocyte aging Expression of SIRT1, 2, 3 mRNA as revealed by real time RT-PCR analysis. The time is 0 h (hours) at 12-14 h of hCG injection. In vitro: samples of 50 MII oocytes were collected after culture for 0, 6, 12, 24 h in M2 medium. In vivo: samples of 50 MII oocytes were collected at 0, 6, 12, 24 h in the oviductal ampullae.*Significantly different (P < 0.05).

To explore the potential involvement of SIRT1, 2, 3 during the oocyte aging process, in vivo and vitro-aged oocytes were collected to analyze the mRNA expression. Notably, results from real-time RT-PCR revealed that SIRT1, 2, 3 mRNA levels significant decreased in MII oocytes aged in vivo or in vitro, when compared to fresh MII oocytes (Figure. 1). Oocytes showed a significant time-dependent decrease in SIRT1, 2, 3 mRNA levels, suggesting that SIRT1, 2, 3 may contribute to mouse postovulatory oocyte aging.

### Inhibition of SIRT1, 2, 3 elevates ROS level in oocytes

**Figure 2:**
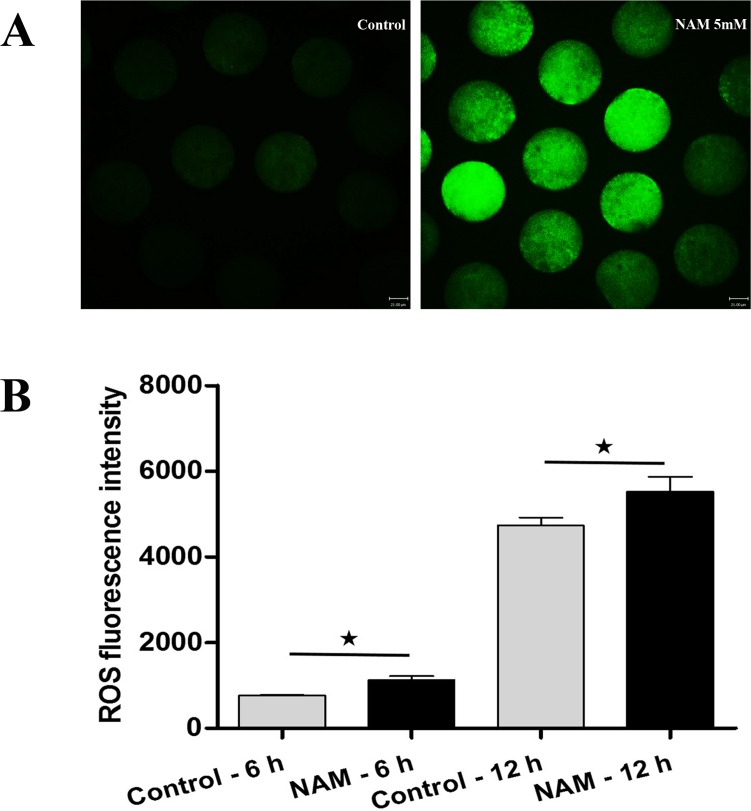
Analysis of ROS production in MII oocytes (**A**) Representative images of carboxy-H2DCF fluorescence in control and NAM-treated oocytes. Scale bar: 21 μm. (**B**) After culture of MII oocytes for 6 h and 12 h in the presence or absence of SIRT1,2,3-nonspecific inhibitor, quantitative analysis of fluorescence intensity was conducted.*Significantly different (P < 0.05).

To investigate whether SIRT1, 2, 3 play a role in protection against oxidative stress, MII oocytes were exposed to Nicotinamide (NAM), a SIRT1, 2, 3 inhibitor. Firstly, ROS level was analyzed at 6 h of oocyte aging in vitro after 0, 1, 5, or 10 mM NAM treatment. When MII oocytes were exposed to 1 mM NAM, the ROS level was not significantly higher compared to untreated oocytes. However, the ROS level was significantly increased by 5 or 10 mM NAM treatment (Figure S1A). Due to the less severe disruption caused by 5 mM NAM, we used this concentration for all subsequent experiments. Subsequen-tly, ROS level was analyzed at 6 h or 12 h of oocyte aging in vitro. Oocytes analyzed at 12 h of aging showed increased ROS levels when compared to oocytes aged for 6 h. When MII oocytes were exposed to 5 mM NAM, the ROS levels observed at 6 h or 12 h were significantly higher compared to that in control untreated oocytes, indicating elevated ROS by SIRT inhibition (Figure 2).

### Inhibition of SIRT1, 2, 3 accelerates MII oocyte aging in vitro

A gradual accumulation of ROS has been considered as a mechanism underlying cell aging. Here we examined whether SIRT1, 2, 3 are involved in MII oocyte aging-associated defects by elevated ROS levels. The occurrence of spindle defects and mitochondrial dys-function in aged MII oocytes has been widely reported in oocyte aging. Therefore, we examined the aging-associated defects in MII oocytes after SIRT modulation.

**Figure 3:**
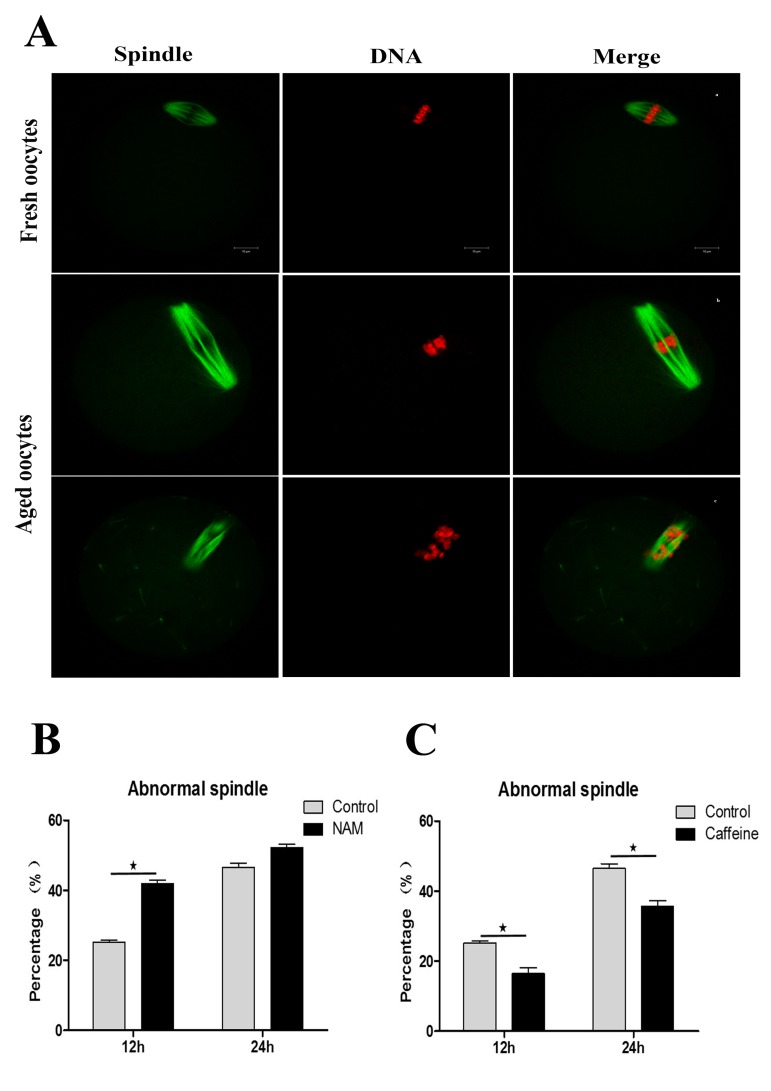
SIRT1, 2, 3 are essential for spindle morphology in aged oocytes (**Aa**) Spindle morphology in fresh MII oocytes. (**Ab**; Ac) Spindle morphology in aged oocytes. DNA (red); Spindle (green). Scale bars: 10 μm. (**B**) Percentages of abnormalities in control or NAM-treated groups at 12h and 24h after aging, respectively. (**C**) The proportion of abnormal spindles in control or caffeine-treated oocytes at 12h and 24h of aging, respectively. Data are expressed as mean ± SEM of at least 3 independent experiments. *Significantly different (P < 0.05).

Firstly, we tested the spindle morphology in fresh, aged, and NAM-treated MII oocytes. As shown in Figure 3A, confocal microscopy revealed that fresh MII oocytes displayed a typical barrel-shaped spindle. However, in aged MII oocytes, spindles became elongated and microtubules became gradually lost from the spindle. Analysis of MII oocytes at 12 h of aging showed that the proportion of abnormal spindles was significantly higher in NAM group than in the control group (41.89 ± 1.66 vs. 25.10 ± 1.26%, P < 0.05). However, the rate of abnormal spindles observed at 24 h of aging showed no significant difference between the NAM group and the control untreated group (52.14 ± 1.88 vs. 46.47 ± 2.34%, P > 0.05).

**Figure 4:**
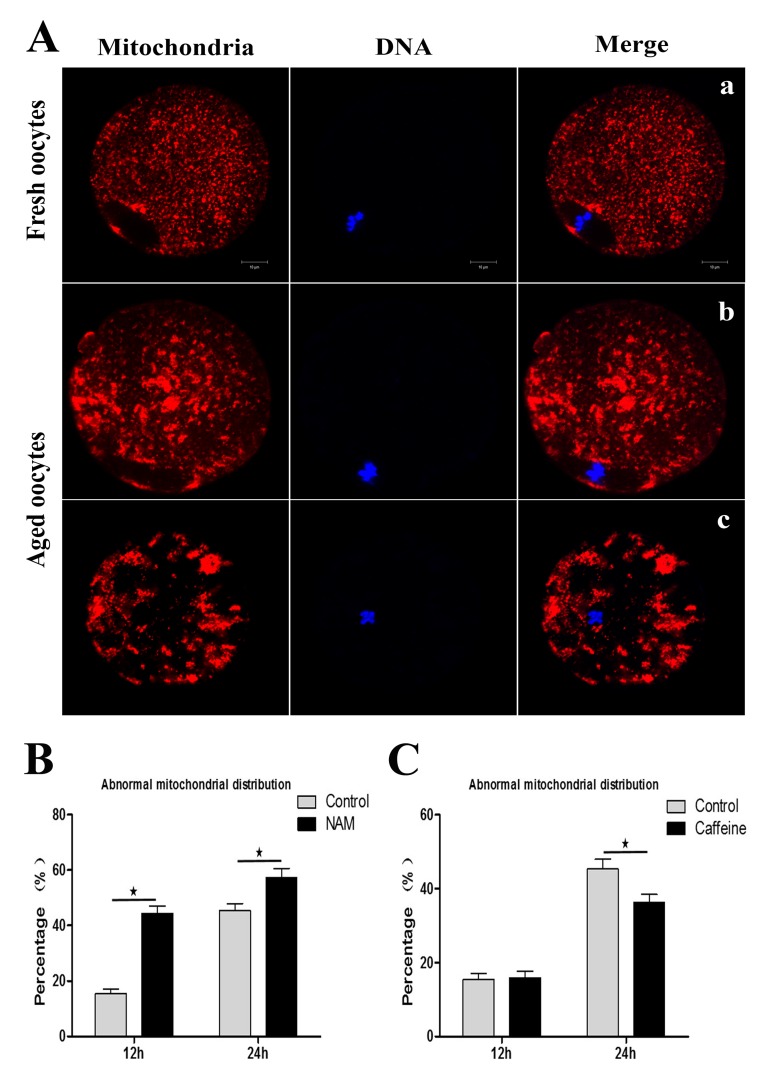
SIRT1, 2, 3 affect mitochondrial distribution during aging of MII oocytes (**Aa**) Distribution of mitochondria in fresh MII oocytes. (**Ab**; **Ac**) Distribution of mitochondrial in aged oocytes. DNA (blue); mitochondria (red). Scale bars: 10 μm. (**B**) At 12 h or 24h of MII oocyte aging, the proportion of the abnormal distribution pattern in control oocytes or NAM-treated oocytes, respectively. (**C**) The proportion of abnormal mitochondrial morphology in control oocytes or caffeine-treated oocytes at 12h and 24h of aging, respectively. Data are expressed as mean ± SEM of at least 3 independent experiments. *Significantly different (P < 0.05).

Mitochondria are also considered to be an important subcellular target of aging-related susceptibility to injury. We next examined whether inhibition of SIRT1, 2, 3 affect mitochondrial distribution during MII oocyte aging. As shown in Figure. 4A, mitochondria displayed a polarized distribution pattern in fresh MII oocytes. In contrast, the distribution of heterogeneous mitochondria in large clusters increased in aged MII oocytes compared to control MII oocytes. At 12 h or 24h of oocyte aging, the proportion of the abnormal distribution pattern was significantly increased in the NAM-treated group when compared with the control group, respectively (44.41 ± 4.66 vs. 15.45 ± 2.91%, P < 0.05; 57.31 ± 5.74 vs. 45.38 ± 4.51%, P < 0.05) (Figure 4B). These results suggested that SIRT1, 2, 3 may play an important role in protecting the structural and functional integrity of the post-ovulatory oocyte.

### Caffeine delays SIRT1, 2, 3 decline and oocyte aging

**Figure 5:**
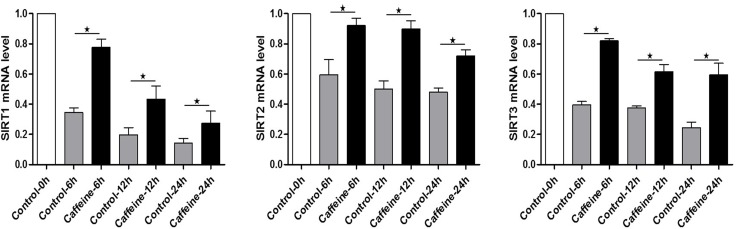
Expression of SIRT1, 2, 3 mRNA in control or caffeine-treated MII oocytes Expression of SIRT1, 2, 3 mRNA as revealed by real time RT-PCR analysis. The time is 0 h at 12-14 h of hCG injection. Samples of 50 MII oocytes were collected after culture for 0, 6, 12, 24 h in the presence or absence of caffeine.*Significantly different (P < 0.05).

Caffeine, as an antioxidant compound, can inhibit hydrogen peroxide (H_2_O_2_)-generation[34]. Previous studies demonstrated that caffeine can delay oocyte aging by reducing p34cdc2 phosphorylation and increasing MPF activity[35]. To explore the potential correlation of caffeine and SIRT1, 2, 3 during the oocyte aging process, caffeine-treated oocytes and control-untreated oocytes were collected to analyze SIRT1, 2, 3 mRNA expression. When MII oocytes were exposed to 10 mM caffeine, the SIRT1, 2, 3 mRNA levels observed at 6 h, 12 h or 24h was significantly higher than that in control untreated oocytes, indicative of the delay of SIRT1, 2, 3 level decline (Figure. 5). Next, we performed caffeine treatment to test whether delaying the SIRT1, 2, 3 decline in aged oocytes could rescue phenotypes of aging. MII oocytes were exposed to caffeine for 12h or 24 h, and then immunolabeled to analyze their spindles and mitochondria. As shown in Figure 3C, oocyte analysis at 12 h or 24h showed that the proportion of aberrant spindles was significantly reduced in the caffeine group compared to the control group (16.46 ± 2.87 vs. 25.10 ± 1.26%, P < 0.05; 35.67 ± 2.74 vs. 46.47 ± 2.34%, P < 0.05). Moreover, these mitochondria-defective phenotypes at 24h were clearly decreased in caffeine-treated oocytes compared to control untreated oocytes (36.34 ± 3.63 vs. 45.38 ± 4.51%, P < 0.05; Figure. 4C). The results suggest that the caffeine-induced delay of SIRT1, 2, 3 decline may prevent postovulatory oocyte aging in vitro.

## DISCUSSION

Oxidative stress is known to continuously threaten the quality of postovulatory oocytes during in vivo processes and in vitro manipulations [36–38]. Early studies have provided evidence for the role of SIRT1, 2, 3 in protection against oxidative stress and aging of oocytes [20, 21, 29, 39]. Here we showed that SIRT1, 2, 3 plays a role in the adaptive response of postovulatory oocytes to oxidative stress, suggesting that SIRT1, 2, 3 prevents postovulatory oocyte aging in MII oocytes aged in vivo or vitro.

Previous studies have shown that SIRT1 and SIRT2 protein or mRNA levels are significantly decreased in aged mouse oocytes [20, 39]. Notably, we found that SIRT1, 2, 3 mRNA expression is down-regulated in MII oocytes aged in vivo or in vitro. Similarly, we also found that compared with fresh oocytes, the level of SIRT1 protein was decreased about 30% at 24 h of MII oocyte aging in vitro (Figure. S1 B; C). These sirtuin proteins have been shown to act as ROS sensors. This led us to explore the link between SIRT1, 2, 3 expression and ROS generation in aged MII oocytes. Thus, ROS levels were analyzed in postovulatory oocytes. We found that ROS levels are markedly increased in oocytes aged for 12h when compared to that of oocytes aged for 6 h, suggesting that ROS may be involved in natural oocyte aging. The SIRT1, 2, 3 inhibitor NAM was used to study the function of SIRT1, 2, 3. The data showed that the level of ROS in NAM treated oocytes was significantly higher than that of control untreated oocytes. These results suggest that SIRT1, 2, 3 may play an important role in ROS clearance during postovulatory oocyte aging in vitro. However, the detailed mechanisms underlying SIRT1, 2, 3 and ROS interaction in postovulatory oocytes deserves further investigation.

In contrast to young oocytes, aged oocytes exhibit increased levels of ROS which implies that the physiological condition of oxidative stress is related to aging. The generation of ROS appears to be associated with the inhibition of SIRT1, 2, 3 in MII oocytes. In order to confirm whether inhibition of SIRT1, 2, 3 can accelerate MII oocyte aging in vitro, we examined the aging-associated index in postovulatory oocytes. Previous studies showed characteristics of aged oocytes[15], which is consistent with our results.

Our data showed that the proportion of spindle defects was significantly higher in the NAM group compared to the control group. SIRT1 was expressed in the cytoplasm of MII oocytes, and mainly associated with the spindle (Figure S1D). At 24h of MII oocyte aging in vitro, SIRT1 disappeared from the spindle position. The fluorescence intensity of SIRT1 was decreased after NAM treatment at 12 or 24h of MII oocyte aging in vitro (Figure. S1E). An intact meiotic spindle is critically important for accurate distribution of chromosomes to the dividing cells. Any errors in this process can lead to the generation of aneuploidy. Thus, our data determined that SIRT1, 2, 3 may have a positive effect on the maintenance of spindles in aged MII oocytes. Mitochondria are considered to be an important subcellular target of oocyte aging. Abnormal mitochondria distribution patterns are frequently found in NAM-treated oocytes, implying that the loss of SIRT1, 2, 3 can accelerate the MII oocyte aging. Subsequently, we also examined the activation susceptibility and oocyte fragmentation. The percentages of the activated oocytes and oocyte fragmentation increased after treatment with NAM (Figure S2 A and C). These data also suggest that inhibition of SIRT1, 2, 3 can accelerate postovulatory aging. Nevertheless, these defective phenotypes of aged MII oocytes were clearly rescued by caffeine treatment.

Moreover, the activation susceptibility and oocyte fragmentation were significantly decreased by caffeine treatment (Figure. S2 B and D). Previous research has identified caffeine as a factor that likely is involved in the delay of oocyte aging [15]. The caffeine delay of postovulatory oocyte aging appears to be dependent on delaying SIRT1, 2, 3 levels. Caffeine can delay oocyte aging by increasing MPF activity. The relationship between MPF activity and delay of SIRTs level decline is worth further investigation. These results suggest that caffeine may be effective in inhibiting the MII oocyte aging process by delaying SIRT1, 2, 3 decline. To investigate whether SIRTs activator has similar function on MII oocyte aging process, we used resveratrol to examine the effects of SIRT1 activators during MII oocyte aging. Compared with caffeine treatment, similar results were observed. Activation of SIRT1 by resveratrol delayed MII oocytes aging in vitro (Figure S2 E and F).

In conclusion, our research demonstrates that the SIRT1, 2, 3 pathway may control ROS generation in postovulatory oocytes and uncovers a potential protective effect of SIRT1, 2, 3 against MII oocyte aging. This is a new area for understanding mechanisms of oocyte aging and assessing postovulatory oocyte quality in a clinical setting.

## MATERIALS AND METHODS

### Oocyte collection

Care and handing of 6-8 week-old ICR mice was conducted in accordance with policies promulgated by the Ethics Committee of the Institute of Zoology, Chinese Academy of Sciences. ICR female mice were administered intraperitoneal injections of PMSG followed 48 h later by hCG to induce superovulation. The superovulated mice were killed 12-14 h after hCG injection and the oviductal ampullae were broken to release the cumulus-oocyte complexes (COCs). The cumulus cells were removed by pipetting in the M2 medium, containing 0.1% hyaluronidase; cumulus-free oocytes were used for the experiments. We set the time 0 hour at 12-14 h of hCG injection. In vitro-aged MII oocytes were collected after culture for 0, 6, 12, 24 h in M2 medium. In vivo-aged MII oocytes were collected at 0, 6, 12, 24 h from the oviductal ampullae.

### Oocyte treatment

Collected oocytes were immediately treated for 12 or 24 h with 5 mM NAM (Cat #S1761, Beyotime) and/or 10 mM caffeine (Cat #C-0750, Sigma, USA) in M2 medium. Oocytes were treated for 12 or 24 h with 2 μM resveratrol (Cat #S1396, Selleck, USA) in M2 medium. The optimal concentration of 5 mM NAM or 10mM caffeine or 2 μM resveratrol was obtained from previously published studies on postovulatory oocyte aging[21, 40, 41]. The MII oocytes were activated by using 10 mM SrCl_2_ in Ca^2+^/Mg^2+^-free CZB.

### Detection of intracellular ROS levels

ROS levels in oocytes were assessed according to the method previously described [20]. The procedure is briefly summarized as follows. Oocytes from different experimental conditions were processed at the same time. Oocytes were incubated for 30 min at 37°C in M2 supplemented with 10 mM carboxy-H2DCF diacetate (Cat #S0033, Beyotime). After washing, oocytes were observed under a spinning disk confocal microscope (Perkin Elmer) with identical settings. Images were analyzed by a Perkin Elmer precisely Ultra VIEW VOX Confocal Imaging System.

### Immunofluorescence analysis

Immunofluorescence was performed as described previously[42]. Briefly, oocytes were fixed in 4% paraformaldehyde in PBS buffer for 30 minutes at room temperature. After being permeabilized with 0.5% Triton X-100 for 20 minutes, they were then blocked in 1% BSA-supplemented PBS for 1 hour at room temperature. For single staining of α-tubulin, oocytes were incubated overnight at 4°C with 1:200 anti-α-tubulin-FITC antibodies (Cat #2125, Beverly, MA). For single staining of Sirt1, oocytes were incubated overnight at 4°C with 1:200 anti-sirt1 antibodies (Cat #AS391, Beyotime), oocytes were incubated with FITC-conjugated goat anti-rabbit secondary antibody for 2 hours at room temperature. DNA was stained with Hoechst 33342 for 15 minutes. For mitochondrial staining, oocytes were incubated for 30 min at 37°C in M2 supplemented with 200 nM MitoTracker Red (Cat #M7512, Invitrogen, USA). After washing, oocytes were stained with Hoechst 33342 (10 mg/ml) for 10 min. Finally, oocytes were mounted on glass slides and viewed under a confocal laser scanning microscope (Zeiss LSM 780).

### Immunoblotting analysis

Immunoblotting was performed as described previously [42]. Briefly, the proteins were separated by SDS-PAGE and then transferred to PVDF membranes. Following transfer, the membranes were blocked in TBST containing 5% BSA for 2 hour at room temperature, followed by incubation overnight at 4°C with mouse anti-Sirt1 antibody (1:1,000) and mouse monoclonal anti-β-actin antibody (1:1,000). The membranes were incubated with 1:1000 HRP-conjugated goat anti-mouse IgG, for 1 hour at 37°C. Finally, the membranes were processed using the enhanced chemiluminescence-detection system (Bio-Rad, CA).

### Real time RT-PCR analysis

Total RNA was extracted from 50 oocytes using RNeasy micro purification kit (Qiagen), the first strand cDNA was generated with M-MLV first strand cDNA synthesis kit (Invitrogen), using oligo (dT) primers. A cDNA fragment of Sirt1, Sirt2 and Sirt3 were amplified. GAPDH was selected as a reference gene. Primer sequences are shown in Table I. The SYBR Premix Ex Tag2 kit (Takara) was used in an ABI prism 7500 Sequence Detection System. Relative gene expression was calculated by the 2^ΔΔCt^ method.

**Table 1:** Primer sequences

	Forward	Reverse
Sirt1	5′-TATCTATGCTCGCCTTGCGG-3′	5′-CGGGATATATTTCCTTTGCAAACTT-3′
Sirt2	5′-TCTGCCACTACTTCATCCGC-3′	5′-ATGTGTAGAAGGTGCCGTGG-3′
Sirt3	5′-TATGGGCTGATGTGATGGCG-3′	5′-AGTCGGGGCACTGATTTCTG-3′
Gapdh	5′-TGGCAAAGTGGAGATTGTTGCC-3′	5′-AAGATGGTGATGGGCTTCCCG-3′

### Statistical analysis

For each experimental series, values are reported as mean ± SEM. Data were generated from at least three per experiment and analyzed by ANOVA using SPSS software (SPSS Inc., Chicago, IL) followed by student-Newman-Keuls test. A P-value of < 0.05 was considered statistically significant.

## SUPPLEMENTARY FIGURES


