# Supplementary material for: SIRT1, 2, 3 protect mouse oocytes from postovulatory aging
Source: Aging (Albany NY). 2016 Mar 10;8(4):685–94. doi: 10.18632/aging.100911 (PMC4925822; doi:10.18632/aging.100911)
Supplement: Supplementary file 1 [file aging-08-685-s001.pdf]

SUPPLEMENTARY FIGURES

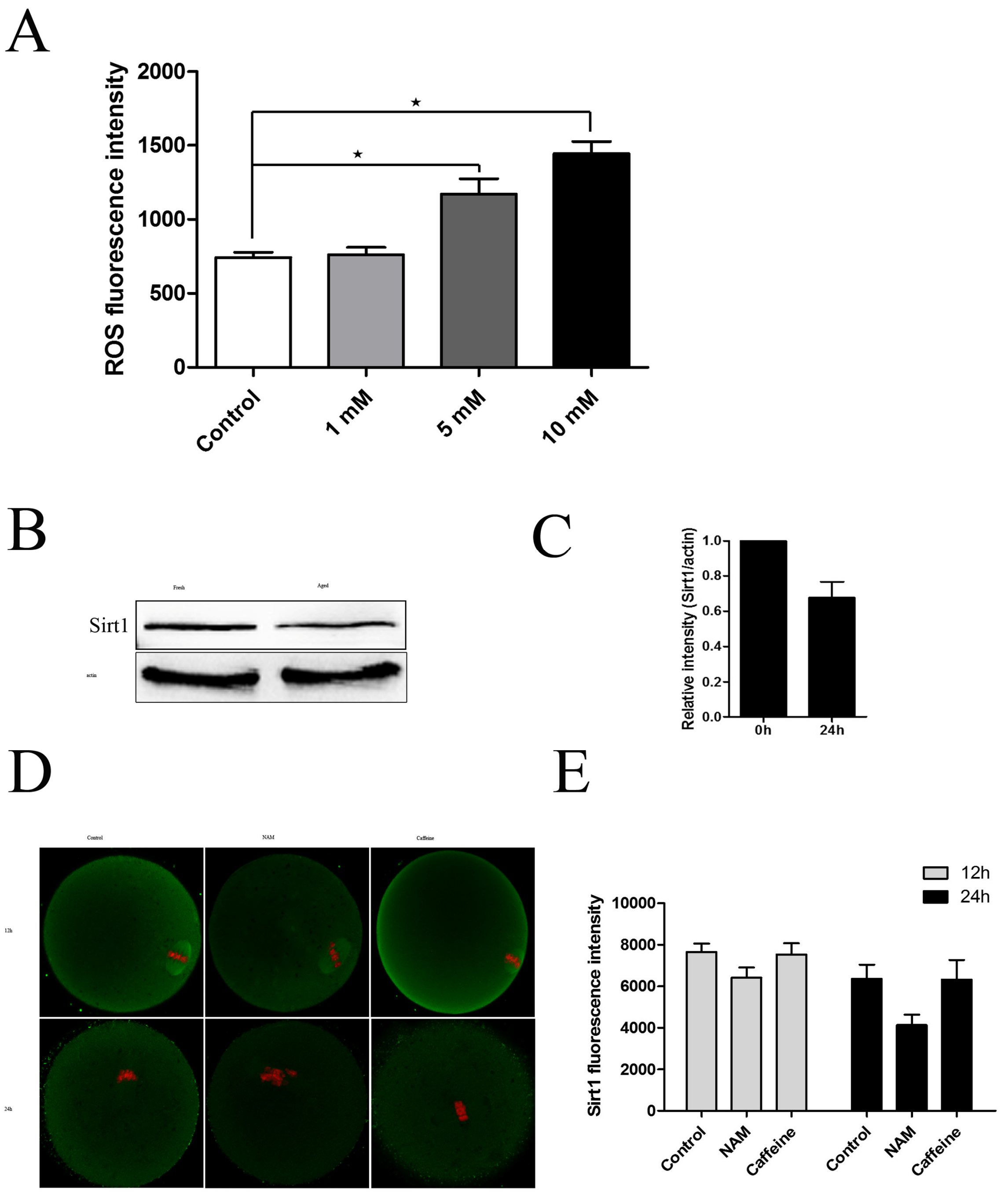

Figure S1. (**A**) Analysis of fluorescence intensity at 6 h of oocyte aging in vitro after 0, 1, 5, or 10 mM NAM treatment. (**B**) The expression of SIRT1 protein in fresh MII oocytes and at 24 h of MII oocyte aging in vitro. (**C**) Quantitative analysis of gray intensity was conducted. (**D**) The sub-cellular localization of SIRT1 after NAM or Caffeine treatment in mouse MII oocytes. (**E**) Quantitative analysis of fluorescence intensity. *Significantly different ($P < 0.05$).

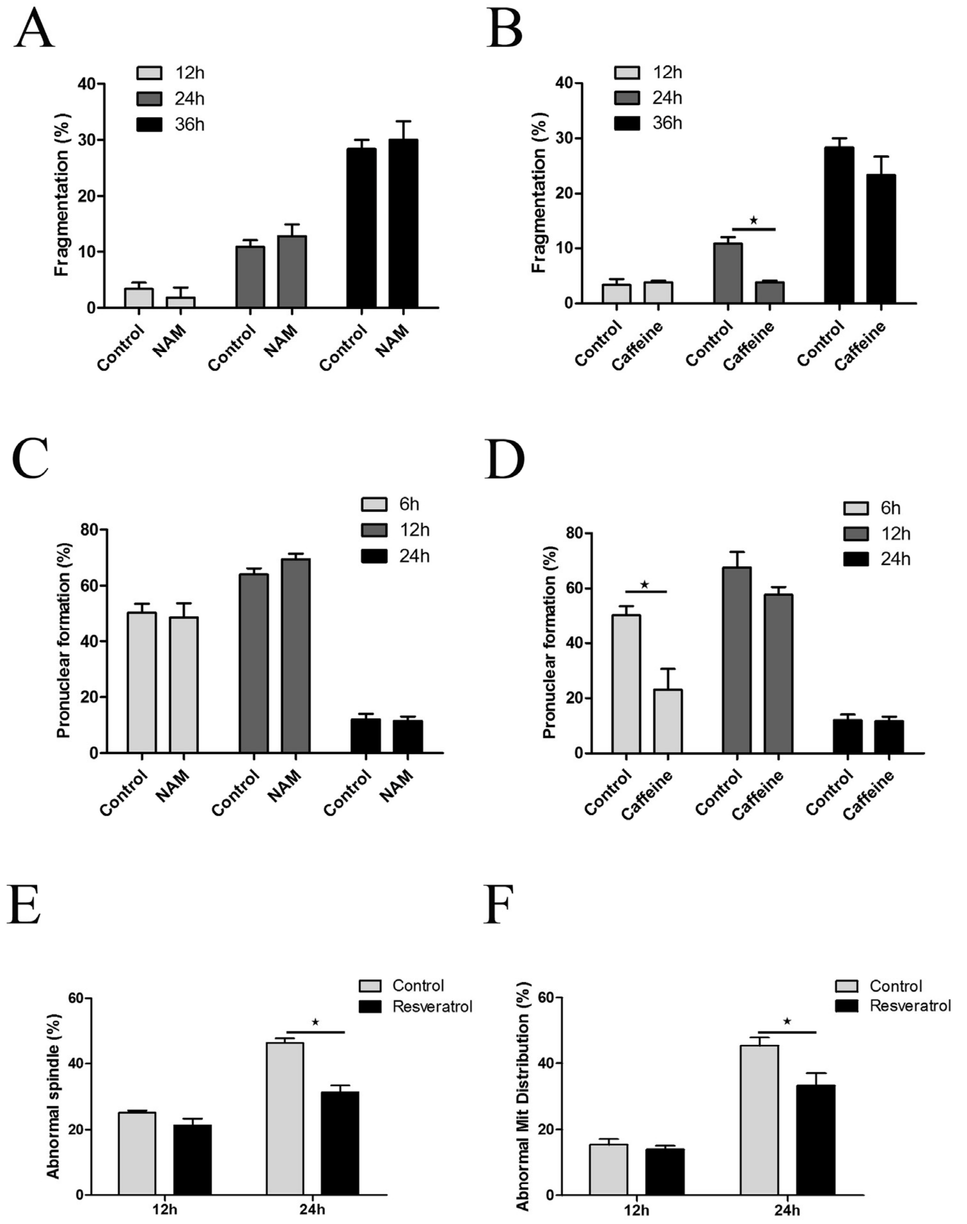

Figure S2. (**A** and **B**) The proportion of oocytes fragmentation in control; NAM-treated and caffeine-treated oocytes at 12 h, 24h and 36h of MII oocyte aging. (**C** and **D**) The proportion of pronuclear formation in control; NAM-treated and caffeine-treated oocytes at 6 h, 14h and 24h of MII oocyte aging. (**E**) Percentages of abnormal spindles in control or resveratrol-treated oocytes at 12h and 24h after aging. (**F**) Percentages of abnormal mitochondrial morphology in control and resveratrol-treated oocytes at 12h and 24h after aging. *Significantly different (P < 0.05).
